# Regulatory Mechanism of *DHCR7* Gene Expression by Estrogen in Chicken Granulosa Cells of Pre-Hierarchical Follicles

**DOI:** 10.3390/biom15050668

**Published:** 2025-05-05

**Authors:** Dandan Li, Longxiao Hu, Qingqing Wei, Li Kang, Yi Sun, Yunliang Jiang

**Affiliations:** 1 College of Animal Science and Technology, Shandong Agricultural University, 7 Panhe Street, Taian 271017, China; 2022010084@sdau.edu.cn (D.L.); 2022110336@sdau.edu.cn (L.H.);; 2 Shandong Provincial Key Laboratory for Livestock Germplasm Innovation & Utilization, Shandong Agricultural University, Taian 271017, China

**Keywords:** chicken follicles, Pre-GCs, DHCR7, estrogen, m^6^A, uORFs

## Abstract

The difference in chicken egg production is closely related to the efficiency of follicle selection, which is marked by granulosa cell differentiation and progesterone production with cholesterol as the substrate. The conversion of 7-dehydrocholesterol to cholesterol catalyzed by 7-Dehydrocholesterol reductase (DHCR7) is the rate-limiting step in cholesterol synthesis. Our previous study revealed that estrogen enhanced the mRNA expression of three *DHCR7* transcript variants (T1, T3, and T4) in a dose-dependent manner in the granulosa cells of chicken pre-hierarchical follicles (Pre-GCs). This study investigates the molecular mechanisms through which estrogen regulates *DHCR7* in chicken Pre-GCs. At the transcriptional level, through CUT&RUN-qPCR, we found that under basal conditions, sterol-regulatory element binding protein 2 (SREBP2) bound to the promoters of three *DHCR7* transcript variants to promote cholesterol synthesis in Pre-GCs to maintain low cholesterol levels; meanwhile upon estrogen treatment, estrogen receptors α and β bound to the regulatory regions of three chicken *DHCR7* transcript variants, leading to a reduction in the interaction between SREBP2 and *DHCR7*. At the translational level, the upstream open reading frames (uORFs) and N6-methyladenosine (m^6^A) modification in the 5′UTR of different *DHCR7* transcripts differentially regulate the expression of T3 and T4, as detected by dual-luciferase reporter assays, but this regulation is not affected by estrogen. This study systematically explores the molecular mechanisms through which estrogen upregulates *DHCR7* expression in chicken Pre-GCs and provides a clue for understanding the molecular mechanisms underlying cholesterol synthesis in chicken ovarian follicles.

## 1. Introduction

Cholesterol homeostasis is critical for organisms, and its dysregulation is associated with various cancers by promoting progression. Cholesterol is a key component of cell membranes and a precursor for steroid hormones, including estrogen and progesterone. It is crucial for cellular functions and signaling [1]. In chickens, follicle selection is a vital process in ovarian development, determining whether follicles can continue to develop and mature to the point of ovulation, thereby directly influencing egg-laying performance and reproductive efficiency [2]. Our previous study revealed a significant increase in the level of cholesterol in chicken granulosa cells after follicle selection [3], highlighting the importance of cholesterol synthesis around follicle selection. 7-dehydrocholesterol reductase (DHCR7) is a critical enzyme in cholesterol biosynthesis, catalyzing the conversion of 7-dehydrocholesterol to cholesterol [4]. Abnormal expression of *DHCR7* has been confirmed to be associated with a range of developmental disorders and metabolic dysfunctions [5,6]. *DHCR7* expression is regulated by sterol-regulatory element binding protein 2 (SREBP2), desmosterol, and cholesterol [7,8].

As a key reproductive hormone, estrogen affects follicle growth and maturation [9].In particular, estrogen promotes the hierarchical development of follicles, the proliferation and differentiation of follicular cells, and the synthesis of steroid hormones [10,11]. In our previous study, through third-generation transcriptome sequencing of chicken follicular granulosa cells before and after follicle selection, we found that *DHCR7* produced three differentially expressed transcript variants (T1, T3, and T4) through 5′ alternative splicing. Additionally, estrogen can dose-dependently modulate the transcription levels of T1, T3, and T4 in the granulosa cells of chicken pre-hierarchical follicles (Pre-GCs) [3]. However, the mechanisms through which estrogen regulates *DHCR7* expression to influence follicle selection remain unresolved.

Recently, the regulation of gene expression at the post-transcriptional and translational levels has attracted increasing attention, with upstream open reading frames (uORFs) and N6-methyladenosine (m^6^A) modifications playing significant roles. uORFs refer to an open reading frame encoded within the 5′ untranslated region (UTR) of an mRNA, which typically regulate the translational efficiency of target genes by competing with the main open reading frame (mORF) during the translation process. The translation of uORFs can lead to ribosome stalling or dissociation, thereby affecting mRNA stability and translational efficiency [12]. Additionally, the presence of uORFs can influence protein synthesis and function through translation signaling [13]. m^6^A is one of the most prevalent epigenetic modifications in mRNA, involving the addition of a methyl group to the nitrogen-6 position of adenosine (A) residues within mRNA molecules. m^6^A modification can influence various aspects of mRNA metabolism, including mRNA stability, splicing, nuclear export, and translational efficiency, thereby modulating gene expression [14]. A recent study reported that m^6^A modification plays a critical role in follicle development and germ cell differentiation [15]. However, whether chicken *DHCR7* expression in Pre-GCs is regulated by uORFs and m^6^A modifications is unknown.

In chickens, the changes in *DHCR7* expression are reported in liver [16] and adipocyte precursor cells [17]; however, its role in ovarian tissues, particularly in chicken follicle development, has not been explored. Therefore, this study aims to investigate the molecular mechanisms through which estrogen upregulates *DHCR7* expression in chicken Pre-GCs on both transcriptional and translational levels, with a focus on how uORF and m^6^A modifications in the 5′UTR influence *DHCR7*’s translational efficiency.

## 2. Materials and Methods

### 2.1. Animals and Sample Collection

In this study, 35-week-old Hy-Line Brown laying hens with at least one month of regular egg production were randomly selected from a local farm affiliated with Shandong Agricultural University. The hens were housed individually in batteries under standard conditions, with free access to feed and water, and maintained under a photoperiod of 16 h of light and 8 h of dark. The hens were slaughtered through cervical dislocation according to the guidelines of the Institutional Animal Care and Use Ethics Committee of Shandong Agricultural University (No. SDAUA-2021-097), and pre-hierarchical follicles were collected and placed in phosphate-buffered saline (PBS) for isolation of granulosa cells.

### 2.2. Culture and Treatment of Granulosa Cells

Pre-GCs were isolated and cultured according to the procedure described by Hu et al. [18]. Briefly, when the cell reached approximately 80% confluency, they were transiently transfected with DNA plasmids using Lipofectamine LTX and Plus Reagent (Thermo Fisher Scientific, Waltham, MA, USA) according to the manufacturer’s instructions. The small interfering RNAs (siRNAs) targeting chicken estrogen receptor 1 (*ESR1*) and estrogen receptor 2 (*ESR2*) were synthesized by Guangzhou RiboBio Co., Ltd., Guangzhou, China, and they were transiently transfected using Lipofectamine RNAiMAX (Thermo Fisher Scientific, Waltham, MA, USA). After 6 h, the medium was replaced by fresh culture medium with or without estradiol (50 nmol/L) and further incubated for 24 h.

### 2.3. Bioinformatics Analysis

Prediction of estrogen receptor α (ERα) transcription factor binding sites in the promoter regions of chicken *DHCR7* was conducted using JASPAR (http://jaspar.genereg.net/, accessed on 11 April 2022) and PROMO (http://alggen.lsi.upc.es/, accessed on 11 April 2022). The uORFs were predicted using the NCBI ORF Finder (https://www.ncbi.nlm.nih.gov/orffinder/, accessed on 10 July 2023) and StarORF (http://star.mit.edu/index.html, accessed on 10 July 2023). m^6^A modification sites on chicken *DHCR7* mRNA were predicted using SRAMP (http://www.cuilab.cn/sramp/, accessed on 18 October 2022).

### 2.4. Plasmid Construction

The 3546 bp upstream sequence of chicken *DHCR7* transcript T1 was inserted into the upstream of the luciferase reporter gene in the pGL3-Basic vector to generate the construct pGL3-T1-F1. Based on the predictions from JASPAR and PROMO, additional constructs were created by making 5′ deletions in pGL3-T1-F1 to generate pGL3-T1-F2 to pGL3-T1-F7, all extending 3′ to the +21 bp relative to the transcription start site (TSS) of T1. With the same method used for T1, the 5′ deletion vectors of transcript T3 and T4 promoter regions were constructed. The difference in the methods is that the 3′ ends of the T3 and T4 constructs extended to +42 bp and +293 bp, respectively. All of the promoter fragments were amplified using Tks Gflex™ DNA Polymerase (TaKaRa, Dalian, China) with genomic DNA of Langya chickens, an indigenous breed in Shandong province, as the template, and all fragments were cloned into the *Kpn* I and *Bgl* II restriction sites of the pGL3-Basic vector. The pGL3-T1-F4 mutation vectors were synthesized by Sangon Biotech (Shanghai) Co., Ltd. The full-length coding sequences of chicken *ESR1* encoding ERα and *ESR2* encoding estrogen receptor β (ERβ) were amplified using TransStart FastPfu DNA polymerase (TransGen, Beijing, China) for the construction of overexpression vectors. *ESR1* was cloned into the *Nhe* I and *Xho* I restriction sites of the pcDNA3.1(+) vector, while *ESR2* was cloned into the *Kpn* I and *Xba* I restriction sites of the pcDNA3.1-3×Flag-C vector. pcDNA3.1-SREBP2-3×Flag was kindly provided by Yanhong Zhang. For the analysis of the 5′UTR, the ATG of humanized Renilla luciferase (hRluc) gene in the psiCHECK2 vector was mutated to TTG and named psiCHECK2M. The wild-type 5′UTR, uORF mutant 5′UTR, and m^6^A binding site mutant 5′UTR were inserted into the *Nhe* I restriction site upstream of the *hRluc* gene in the psiCHECK2M vector. PsiCHECK2M and 5′UTR vectors were synthesized by Sangon Biotech (Shanghai) Co., Ltd. The uORF mutation refers to the alteration of the uORF ATG to the stop codon TGA. All constructs were confirmed through DNA sequencing and restriction enzyme digestion. PCR primer sequences are listed in Supplementary Table S1.

### 2.5. Real-Time Quantitative PCR (RT-qPCR)

For RT-qPCR, total RNA was isolated from Pre-GCs using the RNA simple Total RNA Kit (TIANGEN, Beijing, China). Reverse transcription and RT-qPCR were performed using the Evo M-MLV RT Mix Kit with a gDNA Clean and the SYBR Green Premix Pro Tap HS qPCR Kit (Accurate Biotechnology, Hunan, China), respectively. The 2^−ΔΔCT^ method for calculating the relative gene expression levels was used [19]. For *hRluc* mRNA expression analysis, the luciferase gene was used as the reference, while *GAPDH* was used as the reference for other genes. Primer sequences are listed in Supplementary Table S1.

### 2.6. Protein Extraction and Western Blotting

The total protein was extracted from Pre-GCs using RIPA lysis reagent (Beyotime, Shanghai, China) containing the protease inhibitor (NCM, Suzhou, China) and assessed using a BCA Protein Assay Kit (Beyotime, Shanghai, China). Equal quantities of total protein for each sample were separated using 10% sodium dodecyl sulfate polyacrylamide gel electrophoresis under denaturing conditions and then transferred to a polyvinylidene fluoride membrane (PVDF). Subsequently, the PVDF membrane was blocked in NcmBlot blocking buffer (NCM, Suzhou, China) for 25 min and then blocked with 5% skimmed milk powder for 1.5 h at room temperature. The PVDF membrane was incubated with rabbit anti-ERα polyclonal antibody (ABclonal, Wuhan, China) at 1:1000 dilution, anti-FLAG tag mouse mAb (Engibody, Shanghai, China) at 1:2000 dilution, and mouse anti-GAPDH monoclonal antibody (Proteintech, Wuhan, China) at 1:50,000 dilution. Next, the membrane was incubated with horseradish peroxidase-labeled goat anti-rabbit/anti-mouse IgG (Beyotime, Shanghai, China) at a dilution of 1:4000 for 1 h at room temperature. Finally, protein bands were visualized using BeyoECL Plus (Beyotime, Shanghai, China) in C300 (Azure Biosystems, Dublin, CA, USA). GAPDH was used as the reference protein, and the gray value of the band was quantified using ImageJ 1.46r software.

### 2.7. Dual-Luciferase Reporter Gene Analysis

At 24 h after transfection, cells were washed with PBS and lysed with 100 μL of Passive Lysis Buffer (Promega, Madison, WI, USA). Then, 20 μL of the lysate was used to measure the luciferase activity by using the Dual-Luciferase Assay System according to the manufacturer’s protocol. Firefly luciferase was normalized using renilla luciferase for the pGL3-Basic vector. Renilla luciferase was normalized using firefly luciferase for the psiCHECK2 vector.

### 2.8. CUT&RUN-qPCR

CUT&RUN-qPCR was carried out using the Hyperactive pG-MNase CUT&RUN Assay Kit for PCR/qPCR (Vazyme, Nanjing, China). Briefly, Pre-GCs were incubated with treated ConA Beads Pro for 10 min at room temperature. Next, mouse anti-ERα (ABclonal, Wuhan, China) and mouse IgG antibody (Proteintech, Wuhan, China), or FLAG-tag rabbit mAb (ABclonal, Wuhan, China) and rabbit IgG antibody (CST, Danvers, MA, USA), were added and incubated overnight at 4 °C.

The following day, the pG-Mnase enzyme was added to recognize the antibody and the target protein, and the mixture was incubated at 4 °C for 1 h. Afterward, a CaCl_2_ premix was added to ensure that CaCl_2_ effectively activates Mnase, allowing it to cleave chromatin near the target protein, and incubation continued at 4 °C for 3 h. Next, Stop Buffer was utilized to terminate fragmentation and release the DNA. FastPure gDNA Mini Columns were used to extract DNA, and the final DNA product was used for qPCR analysis.

### 2.9. MeRIP-RT-qPCR

MeRIP assays were conducted with the EpiQuik CUT&RUN m^6^A RNA Enrichment Kit (Epigentek, Farmingdale, NY, USA) following the manufacturer’s instructions. Briefly, m^6^A-modified RNA fragments were pulled down using a beads-bound m^6^A capture antibody. The enriched RNA was subsequently released, purified, and eluted. Finally, the eluted RNA was analyzed through qPCR using the UniPeak U+ One Step RT-qPCR SYBR Green Kit (Vazyme, Nanjing, China) to assess the abundance of m^6^A-modified RNA. The relative m^6^A modification abundance of *DHCR7* transcripts was normalized to the input.

### 2.10. Statistical Analysis

Each experiment was performed with at least three replicates, and for each experimental group, no less than four hens were used. All data were presented as the mean ± standard error of the mean (SEM). Comparisons between two groups were performed using Student’s *t*-test. For comparisons of more than two groups, One-Way ANOVA followed by Tukey’s multiple comparison was performed using SPSS 17.0 (SPSS Inc., Chicago, IL, USA), and *p* < 0.05 was considered significantly different.

## 3. Results

### 3.1. Critical Regulatory Segments of the T1, T3, and T4 Promoters and Regions Responsive to Estrogen Stimulation of DHCR7 in Chicken Pre-GCs

**Figure 1:**
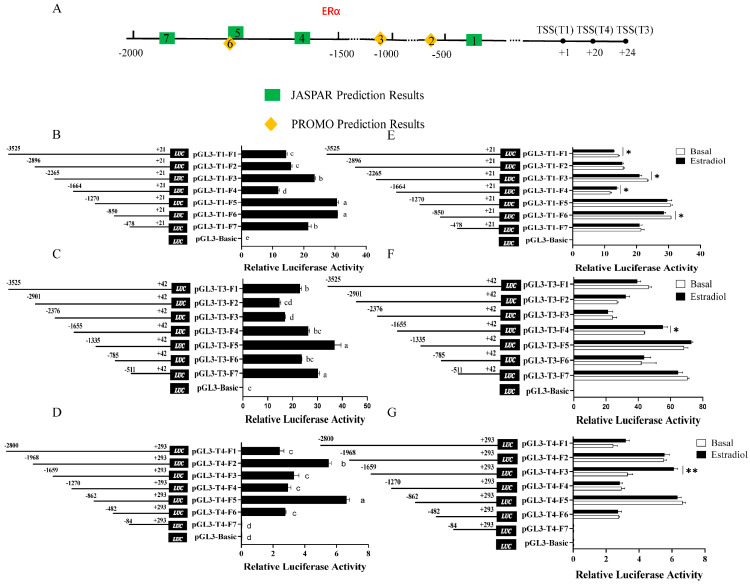
Transcription activity analysis of T1, T3, and T4 and their response to estradiol in chicken Pre-−GCs. (**A**) Predicted estrogen receptor α (ERα) binding sites in the *DHCR7* promoter region. (**B**–**D**) Transcription activity analysis of promoter deletion fragments. (**E**–**G**) Luciferase activity analysis of promoter fragments of different lengths in chicken Pre−GCs after treatment with 50 nmol/L of estradiol. The TSS of T1 is denoted as +1. In Figure 1A, ERα binding sites from 1 to 7 are −307 to −324bp, −601 to −608 bp, −1012 to −1019 bp, −1516 to −1533 bp, −1704 to −1722 bp, −1713 to −1719 bp, and −1758 to −1775 bp. Results are shown as mean ± SEM. * *p* < 0.05, ** *p* < 0.01, and abc *p* < 0.05.

In our previous studies, we revealed that in chicken Pre-GCs, transcripts T1, T3, and T4 of *DHCR7* have different transcription start sites, which lead to distinct promoter regions, and estrogen promoted their expression in a dose-dependent manner [3]. To determine how estrogen exerts its effects, we analyzed the transcriptional regulatory mechanisms of T1, T3, and T4 in chicken Pre-GCs. Four potential estrogen receptor α binding sites in the promoters of T1, T3, and T4 were predicted using JASPAR, while three sites were predicted using PROMO (Figure 1A). Dual luciferase reporter constructs containing different fragments of T1, T3, and T4 promoter were transfected into chicken Pre-GCs. A significant decrease in luciferase activity was observed when the T1 promoter regions were deleted from −2265 bp to −1664 bp and −850 bp to −478 bp (Figure 1B), indicating the presence of positive *cis*-acting elements within these regions. In contrast, the luciferase activity of regions from −2896 bp to −2265 bp and −1664 bp to −1270 bp in T1 (Figure 1B) significantly increased, suggesting that these regions contain negative *cis*-acting elements. Similarly, positive *cis*-acting elements exist in the T3 promoter regions from −3525 bp to −2901 bp and −1335 bp to −785 bp (Figure 1C) and in the T4 promoter regions from −1968 bp to −1659 bp and −862 bp to −482 bp (Figure 1D). Conversely, the promoter regions from −2376 bp to −1335 bp and −785 bp to −511 bp in T3 (Figure 1C) and −2800 bp to −1968 bp and −1270 bp to −862 bp in T4 (Figure 1D) contain negative *cis*-acting elements.

After treatment with 50 nmol/L of estradiol, the luciferase activity of pGL3-T1-F4 (Figure 1E), pGL3-T3-F4 (Figure 1F), and pGL3-T4-F3 (Figure 1G) vectors in chicken Pre-GCs significantly increased, suggesting that the regions from −1664 bp to −1270 bp in T1, −1655 bp to −1335 bp in T3, and −1659 bp to −1270 bp in T4 contain estrogen-responsive elements that might interact with certain estrogen receptors (ERs).

### 3.2. ER and SREBP2 Promote the Expression of DHCR7 by Binding to the Promoter Regions of T1, T3, and T4 in Chicken Pre-GCs

**Figure 2:**
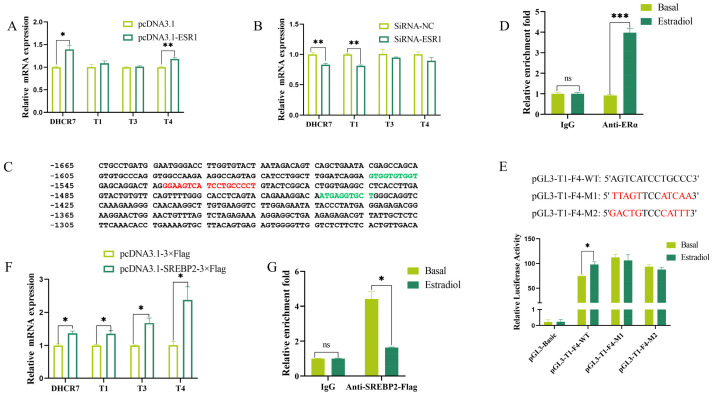
Binding of ERα and SREBP2 to the promoter regions of the three *DHCR7* transcripts in chicken Pre-GCs. Effects of *ESR1* and SREBP2 overexpression (**A**,**F**) and *ESR1* knockdown (**B**) on the expression levels of *DHCR7*, T1, T3, and T4. (**C**) Sequence analysis of estrogen response region. Red font represents the predicted ERα binding site, while green font represents the predicted SREBP2 binding sites. (**D**,**G**) CUT&RUN-qPCR validation of ERα and SREBP2 binding to the promoter regions of *DHCR7*. (**E**) The effect of the ERE on pGL3-T1-F4 response to 50 nmol/L of estradiol. WT, AGTCATCCTGCCC; M1, TTAGTTCCATCAA; M2, GACTGTCCCATTT. In (**E**), mutation sites are labeled in red. Results are shown as mean ± SEM. * *p* < 0.05, ** *p* < 0.01, and *** *p* < 0.001.

To confirm whether estrogen promotes chicken *DHCR7* transcription through ER, we firstly transfected the Pre-GCs with *ESR1* and *ESR2* overexpression plasmids and siRNAs targeting *ESR1* and *ESR2* (Figures S1A–F and S2A–C), respectively. Overexpression of *ESR1* significantly increased the total mRNA expression of *DHCR*7 and the expression of T4 (by approximately 25% and 15%, respectively) (Figure 2A). However, knockdown of *ESR1* significantly decreased the total mRNA expression of *DHCR7* and the expression of T1 (by approximately 16% and 19%, respectively) (Figure 2B). RT-qPCR showed that *ESR2* had no significant effect on the mRNA expression of *DHCR7* and the three transcripts (Figure S2D,E).

By analyzing estrogen response regions, we found that the regions from −1664 bp to −1270 bp in T1, −1655 bp to −1335 bp in T3, and −1659 bp to −1270 bp in T4 contain one estrogen response element (ERE) (Figure 2C). Then, to confirm the direct involvement of ERs, a CUT&RUN assay was performed. The results showed that ERα and ERβ bound to pGL3-T1-F4, pGL3-T3-F4, and pGL3-T4-F3 (Figure 2D and Figure S2F). After mutating this ERE, pGL3-T1-F4 no longer responded to estradiol stimulation (Figure 2E). These results collectively indicate that in chicken Pre-GCs, ER promotes the transcription of the three *DHCR7* transcripts by binding to the ERE in their respective promoter regions.

The expression of *DHCR7* in humans and mice is regulated by the SREBP2 transcription factor [7,20]. Using the JASPAR tool, two binding sites for the SREBP2 transcription factor were predicted in the estrogen response region of chicken *DHCR7* promoter (Figure 2C, green part). Overexpression of the mature form of SREBP2 (Figure S3A,B) significantly promoted the mRNA expression of *DHCR7* and its three transcript variants in chicken Pre-GCs (Figure 2F). Estradiol treatment reduced the binding of SREBP2 to chicken *DHCR7* at a concentration of 50 nmol/L (Figure 2G).

### 3.3. Genetic Polymorphism of DHCR7 and Its Response to Estrogen in Chicken Pre-GCs

**Figure 3:**
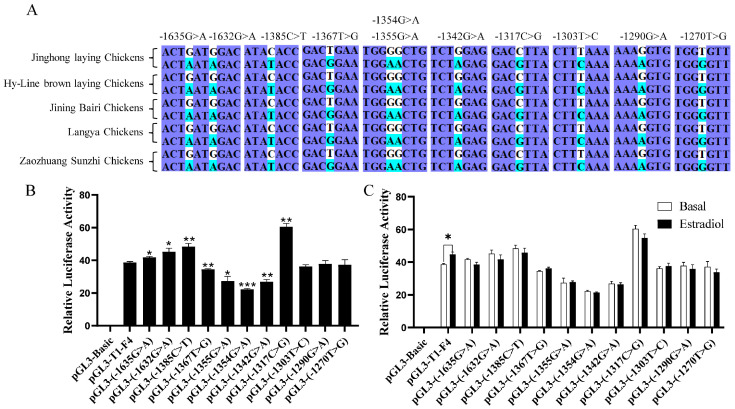
Analysis of *DHCR7* polymorphisms and estrogen response in chicken Pre-GCs. (**A**) Analysis of polymorphisms in the estrogen-responsive region. Potential SNPs analyzed for their effect on promoter activity (**B**) and their response to 50 nmol/L of estradiol (**C**). Results are shown as mean ± SEM. * *p* < 0.05, ** *p* < 0.01, and *** *p* < 0.001.

Combined with the estrogen-responsive region previously found (Figure 2C), 11 potential single nucleotide polymorphism (SNP) sites were identified in Jinghong laying chickens, Hy-Line Brown laying chickens, Jining Bairi chickens, Langya chickens, and Zaozhuang Sunzhi chickens (Figure 3A). After mutating these sites, the luciferase activities at eight sites significantly changed (Figure 3B). However, none of these SNPs exhibits significant differences upon treatment with estradiol in chicken Pre-GCs (Figure 3C).

### 3.4. Effects of 5′UTRs on DHCR7 Expression in Chicken Pre-GCs

**Figure 4:**
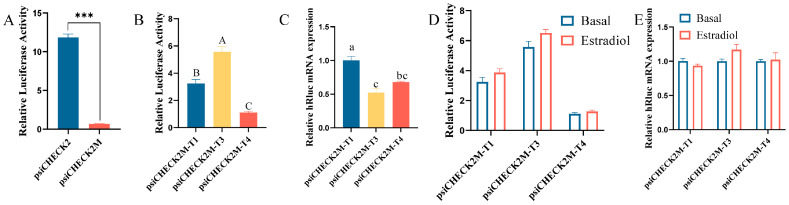
Effects of 5′UTR on the expression of *DHCR7* in chicken Pre-GCs. (**A**) Validation analysis of the psiCHECK2M reporter gene vector. Dual-luciferase (**B**) and RT-qPCR (**C**) analyses of the translational efficiency of T1, T3, and T4. (**D**,**E**) The effect of 50 nmol/L of estradiol on the translational efficiency of T1, T3, and T4. The 5′UTRs of T1, T3, and T4 were inserted into psiCHECK2M and denoted as psiCHECK2M-T1, psiCHECK2M-T3, and psiCHECK2M-T4, respectively. The results are shown as mean ± SEM. abc, *p* < 0.05; ABC, *p* < 0.01; and ***, *p* < 0.001.

Three chicken *DHCR7* transcripts of T1, T3, and T4 have the same coding sequence (CDS) but different 5′UTRs (Figure S4). We next analyzed the effect of 5′UTRs on the translational efficiency of *DHCR7* and its response to estrogen. To achieve this, we constructed a psiCHECK2M vector, which showed decreased translation activity compared to the psiCHECK vector (Figure 4A), and we inserted different 5′UTR fragments into it to assess their impact on translational efficiency and estrogen responsiveness. We found that the T3 5′UTR had the strongest promoting effect on hRluc gene protein expression, followed by T1 and T4 (Figure 4B), while the T1 5′UTR had the strongest promoting effect on its mRNA level, followed by T4 and T3 (Figure 4C). No response to estradiol in terms of the hRluc gene protein and mRNA expression was observed (Figure 4D,E).

### 3.5. Effect of uORFs in 5′UTR on DHCR7 Expression in Chicken Pre-GCs

**Figure 5:**
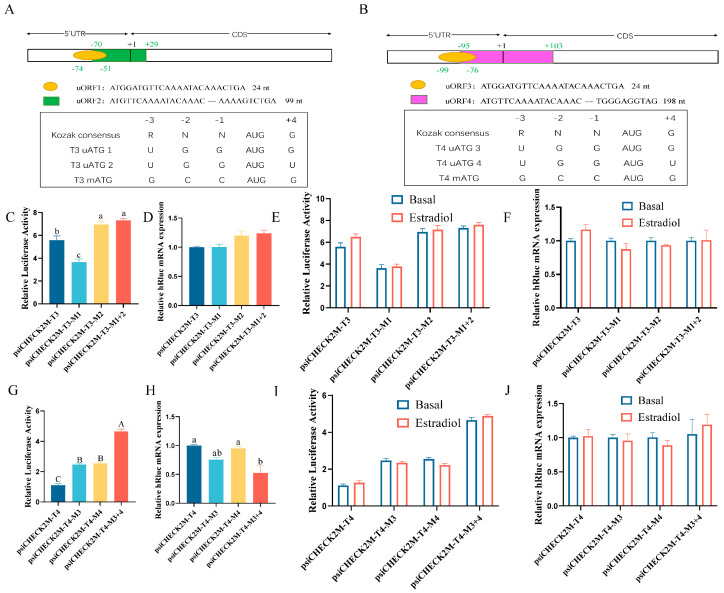
The effects of uORFs on the expression of *DHCR7* in chicken Pre−GCs. (**A**,**B**) Prediction analysis of uORFs in the 5′UTR of T3 and T4. Dual−luciferase (**C**,**G**) and RT−qPCR (**D**,**H**) analyses of the impact of uORFs in the 5′UTRs of T3 and T4. (**E**,**F**,**I**,**J**) Effect of 50 nmol/L of estradiol on the function of uORFs. uATG, upstream ORF ATG; mATG, main ATG. For T3 and T4, the first nucleotide (**A**) of the start codon is denoted as +1. Results are shown as mean ± SEM. abc, *p* < 0.05; ABC, *p* < 0.01.

To explore the potential mechanisms through which the three *DHCR7* transcripts differ in translational efficiency in chicken Pre-GCs, we first analyzed their 5′UTRs (Figure S4). Both T3 and T4 are predicted to have two overlapping uORFs (Figure 5A,B). Analysis of the Kozak sequence strength around the ATG of these uORFs and the main ATG revealed that both T3 mATG and T4 mATG have a stronger Kozak sequence, indicating greater translational efficiency (Figure 5A,B). The Kozak sequence strength is crucial, as it influences the initiation efficiency of translation, affecting the role of uORFs. While the sequence around T3 uATG1 and T4 uATG3 generally conforms to the Kozak rule, those around T3 uATG2 and T4 uATG4 do not (Figure 5A,B).

Given that uORFs can regulate the translational efficiency of downstream genes, we next analyzed the impact of uORFs on *DHCR7* expression in chicken Pre-GCs. We found that the uORF1 (M1) mutation reduced the hRluc protein expression but had no effect on *hRluc* mRNA expression (Figure 5C,D); however, the uORF2 (M2) mutation and the mutation of both uORF1 and uORF2 (M1 + 2) promoted hRluc protein expression but had no effect on *hRluc* mRNA (Figure 5C,D). Therefore, we concluded that the uORF1 (M1) mutation promotes the translational efficiency of T3, while the uORF2 (M2) mutation alone or in combination with uORF1 (M1 + 2) suppresses the translational efficiency of T3. When either uORF3 (M3) or uORF4 (M4) was mutated, hRluc protein expression was inhibited, and a stronger inhibitory effect was observed when both uORF3 and uORF4 (M3 + 4) were mutated simultaneously (Figure 5G). Additionally, mutation of both uORF3 and uORF4 (M3 + 4) inhibited *hRluc* mRNA expression, while mutation of uORF3 (M3) or uORF4 (M4) alone had no effect on *hRluc* mRNA levels (Figure 5H). Therefore, we concluded that both uORF3 and uORF4 in T4 suppress the translational efficiency and have a cumulative effect. As expected, the addition of estradiol had no significant effect on the function of the uORFs in the 5′UTR of T3 and T4 transcripts (Figure 5E,F,I,J).

### 3.6. Effect of m^6^A Modification in 5′UTR on DHCR7 Expression in Chicken Pre-GCs

**Figure 6:**
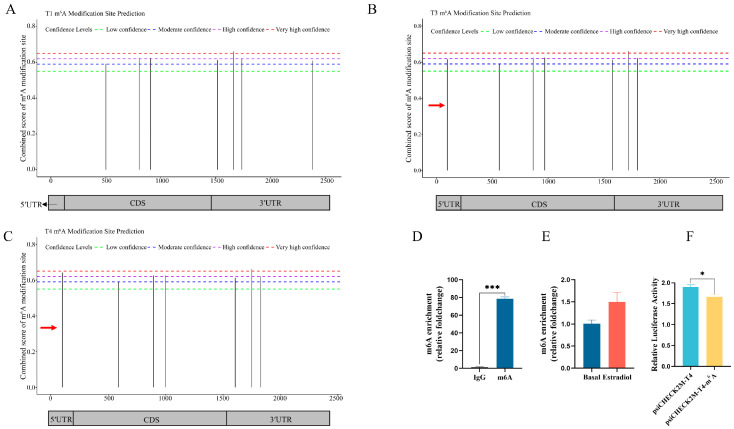
The effects of m^6^A modification on the expression of *DHCR7* in chicken Pre-GCs. (**A**–**C**) Prediction results of m^6^A binding sites from SRAMP. Red arrows indicate the regions within the 5′UTR of T3 and T4 transcripts with m^6^A modifications. (**D**) Detection of m^6^A modification in the 5′UTR of T4 in Pre-GCs. (**E**) Effect of 50 nmol/L of estradiol addition on m^6^A abundance in Pre-GCs. (**F**) Comparison of luciferase activity between wild-type and mutant m^6^A binding site plasmids. psiCHECK2M-T4-m^6^A is the mutant of the m^6^A binding site GGACT to GGCCT. Results are shown as mean ± SEM. * *p* < 0.05 and *** *p* < 0.001.

m^6^A modification in 5′UTR can influence the translational efficiency of mRNA. To determine the existence of m^6^A modification in the 5′UTR of chicken *DHCR7*, we predicted and detected the enrichment of the potential m^6^A modification sites in Pre-GCs and showed that there is one site in T3 with moderate confidence and another site in T4 with very high confidence (Figure 6A–C and Supplementary Table S2). The 5′UTR of T4 was m^6^A-modified (Figure 6D), but estradiol treatment did not significantly enhance its m^6^A abundance in Pre-GCs (Figure 6E). To further demonstrate the essential role of m^6^A modification in the regulation of *DHCR7*, we constructed a luciferase reporter named psiCHECK2M-T4-m^6^A by mutating the potential m^6^A motif in the 5′UTR of T4. The dual-luciferase assay showed that the translational efficiency of *DHCR7* was significantly reduced after transfection of the psiCHECK2M-T4-m^6^A vector (Figure 6F), showing that the m^6^A modification in the T4 5′UTR facilitates the translation of T4.

## 4. Discussion

In the ovaries of poultry, such as chickens, Pre-GCs are in an undifferentiated state and convert only small amounts of cholesterol to progesterone; meanwhile, after follicle selection, granulosa cells of hierarchical follicles (Post-GCs) begin to differentiate and synthesize large amounts of progesterone [21,22]. D*HCR7* is a key and rate-limiting enzyme in cholesterol synthesis and thus may regulate the level of cholesterol required for the production of steroid hormones, such as progesterone. We previously found that after chicken follicle selection, the levels of both of the three *DHCR7* transcripts and cholesterol significantly increased, and the expression of mRNA for all three D*HCR7* transcripts was promoted by estrogen in a dose-dependent manner in Pre-GCs [3]. In this study, the mechanisms underlying the upregulation of *DHCR7* by estrogen in chicken Pre-GCs were analyzed at the transcriptional and translational levels.

At the transcriptional level, bioinformatics analysis predicted multiple EREs in the *DHCR7* promoter region. Through experimental validation, we identified a functional ERE within the estrogen-responsive region of chicken *DHCR7*. ERα and ERβ can bind to this response region and are no longer able to respond to estrogen stimulation when this ERE is mutated, demonstrating that this ERE is functional and that ERs bind directly to this ERE in chicken *DHCR7*. Notably, the diversity of ERE sequences is considerable, with most EREs differing from the canonical ERE [23], which suggests that other ER binding sites might exist in chicken *DHCR7*. Regulation of target gene expression by estrogen through ERE is its classical mode of action. However, an increasing number of studies indicate that estrogen can also regulate the expression of target genes through other pathways. For example, ERs can interact with ERE half sites (ERE 1/2, referring to either the proximal or distal half of a palindromic sequence) to exert their effects [23,24,25]. Using the PROMO tool, three half-sites in the chicken *DHCR7* promoter region (Figure 1A) were predicted, the functions of which require further investigation. The ERs can also interact with transcription factors, such as SP1 and AP1, to achieve regulation of target genes. In human T47D cells, regulation of human prolactin by estrogen is dependent on the synergistic interaction of the ERE and AP1 sites [26]. In response to estrogen stimulation, ERα/SP1 forms a complex that induces low-density lipoprotein receptor gene expression [27]. However, we did not find chicken-specific SP1 or AP1 in the commonly used transcription factor prediction databases. Given the high conservation of transcription factor binding sites across species, SP1 and AP1 binding sites in the chicken *DHCR7* promoter region were predicted using human and mouse species as references, and potential SP1 and AP1 binding sites were identified in the chicken *DHCR7* promoter.

When intracellular cholesterol levels are low, the SREBP cleavage-activating protein (SCAP) undergoes a conformational change, leading to the transportation of the SREBP2-SCAP complex from the endoplasmic reticulum to the Golgi apparatus. Subsequently, the nuclear form of SREBP2 is released, enters the nucleus, and activates the transcription of target genes [28]. When intracellular cholesterol levels rise, cholesterol binds to SCAP, promoting the interaction between SCAP and the insig protein on the endoplasmic reticulum membrane, thereby preventing the transportation of the complex and inhibiting the binding of SREBP2 to target genes [29]. It is likely that in the absence of estrogen, SREBP2 binds to the *DHCR7* promoter region to maintain low cholesterol levels in Pre-GCs; however, when estrogen is added, estrogen receptors bind to the *DHCR7* promoter, increasing the expression of *DHCR7* and intracellular cholesterol levels and reducing the binding of SREBP2.

At the translational level, we first investigated the impact of different 5′UTRs on the translational efficiency of *DHCR7* and found significant differences in the translational efficiency among the three transcripts in chicken Pre-GCs. Although the coding region is identical, the different 5′UTRs of the three transcripts produced different effects on *DHCR7* protein expression by regulating the translational efficiency in chicken Pre-GCs. To further explore the causes of the differences in translational efficiency among T1, T3, and T4, we analyzed the role of uORFs. We found that uORF1 in T3 promotes translational efficiency, while uORF2 in T3 suppresses translational efficiency, and uORF3 and uORF4 in T4 inhibited translational efficiency with a cumulative effect in chicken Pre-GCs. Generally, uORFs inhibit the translational efficiency of the mORF, and only a few uORFs enhance the translational efficiency of mORFs [12,30,31]. During the differentiation of monocytes into macrophages, uORFs suppress the expression of the TNFAIP2 protein in monocytes, but they are inactivated in mature macrophages, allowing for the massive expression of TNFAIP2 protein [32]. In rice, uORFs in *OsNLA1* can promote its phosphate transport and reproduction [31]. uORFs may affect the translation of the main coding region of a gene through numerous mechanisms, including leaky scanning, ribosome stalling, and translation reinitiation [33]. We speculate that in chicken Pre-GCs, the strong promotion of protein expression by the T3 5′UTR is likely caused by its specific sequence structure or the presence of uORFs, thus promoting efficient ribosome binding and translation initiation, while the two uORFs present in the T4 5′UTR may reduce translational efficiency by inhibiting effective ribosomal scanning or promoting ribosomal stalling, which requires further investigation. The function of uORFs in controlling *DHCR7* translation is not reported in other species.

Additionally, the presence of the m^6^A binding site within the 5′UTR of T4 further affects the translational efficiency of chicken *DHCR7* in Pre-GCs. Although two uORFs within the 5′UTR of T4 suppress translational efficiency in chicken Pre-GCs, m^6^A modifications can partially counteract this inhibitory effect by promoting cap-independent translation initiation and enhancing ribosome recruitment [34]. This suggests that multiple regulatory elements within the same 5′UTR co-regulate gene expression through complex and dynamic mechanisms, with the specific outcomes depending on the interactions and relative strengths of these elements [35]. Similarly, in bladder cancer tissues, the upregulation of *DHCR7* expression is induced by m^6^A modification, which changes mRNA stability and translational efficiency [36]. m^6^A regulates CTNNB1 protein expression by altering the stability of mRNA [37]. Estrogen promotes m^6^A modification and enhances translational efficiency by inhibiting the expression of demethylase of m^6^A modification [38].

Previous studies indicate that the DNA sequences corresponding to the 5′UTRs of many genes possess promoter activity, such as the 5′UTRs of the V1b vasopressin receptor [39] and the *WNK8* gene [40]. Estrogen can regulate the expression of target genes both at transcriptional and translational levels [41]. Although the function of uORFs is highly dependent on environmental factors [42], the 5′UTR of chicken *DHCR7* did not respond to estrogen stimulation regardless of whether the uORFs were mutated. We propose three possibilities for this observation. Firstly, the 5′UTR of the chicken *DHCR7* gene may lack estrogen receptor binding sites, or the existing binding sites may have low activity, preventing estrogen from working. Secondly, estrogen may primarily regulate chicken *DHCR7* expression at the transcriptional level rather than the translational level [41,43]. Finally, in chickens, other post-transcriptional regulatory mechanisms, such as mRNA subcellular localization or post-translation modifications, may play a more significant role in the response of *DHCR7* to estrogen. Further studies are needed to validate these hypotheses, such as identifying potential ER binding sites in the 5′UTR or exploring the role of post-transcriptional regulators in estrogen signaling.

## 5. Conclusions

In summary, in this study, we revealed that under basal conditions, SREBP2 bound to the promoters of three *DHCR7* transcript variants to promote cholesterol synthesis in Pre-GCs to maintain low cholesterol levels; meanwhile, upon estrogen treatment, estrogen receptors α and β bound to the regulatory regions of three chicken *DHCR7* transcript variants, leading to a reduction in the interaction between SREBP2 and *DHCR7*. Furthermore, the different 5′UTRs of chicken *DHCR7* transcripts exhibit significant variations in translational efficiency. The uORFs and m^6^A modifications in 5′UTR differentially regulate the expression of T3 and T4, but this regulation is not influenced by estrogen. These results provide some clues for a deeper understanding of the molecular regulatory mechanisms of *DHCR7* in chicken ovarian development by estrogen. Studies on *DHCR7* in vertebrates have mostly focused on the effects of gene mutations, with few studies reporting on the regulation of *DHCR7* expression. The findings of this study also serve as a reference for elucidating the role and regulatory mechanisms of DHCR7 in other vertebrate species.

## Data Availability

The data presented in this study are available in this article (and the supplementary material).

## Supplementary Materials

The following supporting information can be downloaded at https://www.mdpi.com/article/10.3390/biom15050668/s1, Figure S1: ESR1 was successfully overexpressed and knocked down in chicken Pre-GCs; Figure S2: Binding of ERβ to the promoter regions of the three *DHCR7* transcripts in Pre-GCs; Figure S3: SREBP2 was successfully overexpressed in chicken Pre-GCs; Figure S4: Structure and 5′UTR region differences of *DHCR7* transcripts; Table S1: Primer sequences used in the present study; Table S2: SRAMP prediction results of *DHCR7* m^6^A sites.

## Abbreviations

The following abbreviations are used in this manuscript:

*DHCR7*	7-Dehydrocholesterol reductase
T1, T3 and T4	Three DHCR7 transcript variants
SREBP2	Sterol-regulatory element binding protein 2
uORFs	Upstream open reading frames
m^6^A	N6-methyladenosine modification
Pre-GCs	Granulosa cells of pre-hierarchical follicles
5′UTR	5′ untranslated region
mORF	Main open reading frame
PBS	Phosphate-buffered saline
siRNAs	Small interfering RNA
ESR1	Estrogen receptor 1
ESR2	Estrogen receptor 2
ERα	Estrogen receptor α
ERβ	Estrogen receptor β
*hRluc*	Humanized Renilla luciferase
PVDF	Polyvinylidene fluoride
SEM	Standard error of the mean
ER	Estrogen receptor
TSS	Transcription start site
ERE	Estrogen response element
SNP	Single nucleotide polymorphism
CDS	Coding sequence
Post-GCs	Granulosa cells of hierarchical follicles
SCAP	SREBP cleavage-activating protein
