# Supplementary material for: Regulatory Mechanism of DHCR7 Gene Expression by Estrogen in Chicken Granulosa Cells of Pre-Hierarchical Follicles
Source: Biomolecules. 2025 May 5;15(5):668. doi: 10.3390/biom15050668 (PMC12108600; doi:10.3390/biom15050668)
Supplement: Supplementary file 1 [file biomolecules-15-00668-s001.zip › biomolecules-3601154-supplementary.pdf]

Supplemental Figures

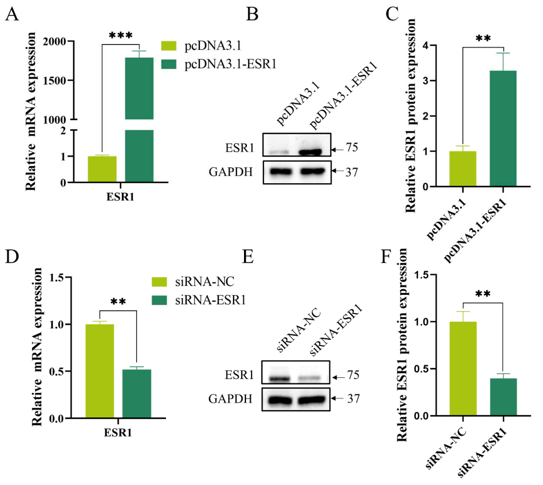

Supplemental Fig. S1. *ESR1* was successfully overexpressed and knocked down in chicken Pre-GCs. RT-qPCR and Western blot analyses were conducted to verify *ESR1* overexpression (A-C) and knockdown (D-F) effects. Results are shown as mean±SEM. ***p* <0.01, and ****p* <0.001.

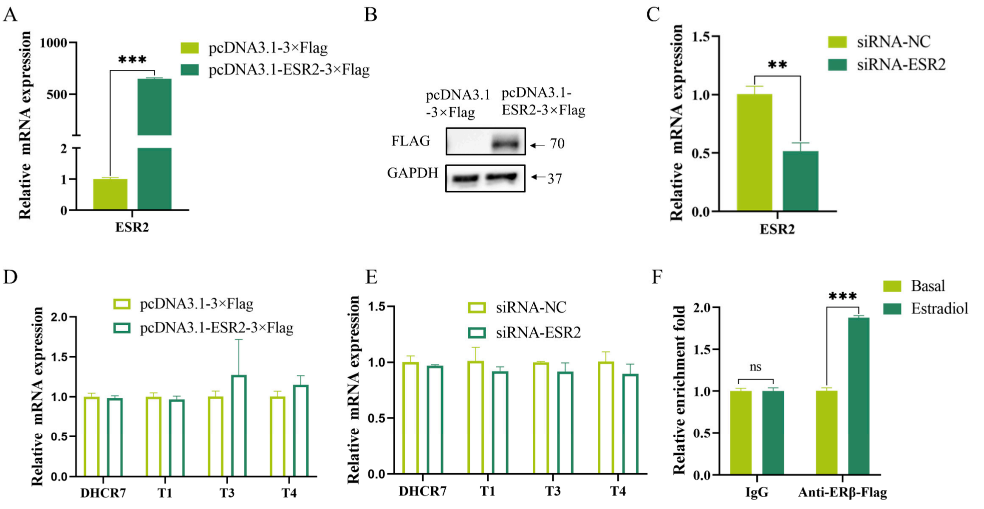

Supplemental Fig. S2. Binding of ERβ to the promoter regions of the three *DHCR7* transcripts in

Pre-GCs. Effects of *ESR2* overexpression (A-B, D) and knockdown (C, E) on the expression levels of *ESR2*, *DHCR7*, T1, T3 and T4. (F) CUT&RUN-qPCR validation of ERβ binding to the promoter regions of *DHCR7*. Results are shown as mean±SEM. $**p < 0.01$, and $***p < 0.001$.

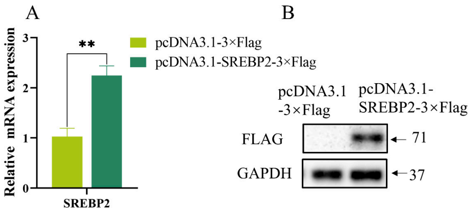

Supplemental Fig. S3. SREBP2 was successfully overexpressed in chicken Pre-GCs. RT-qPCR and Western blot analyses were conducted to verify SREBP2 overexpression (A-B) effects. Results are shown as mean±SEM. $**p < 0.01$.

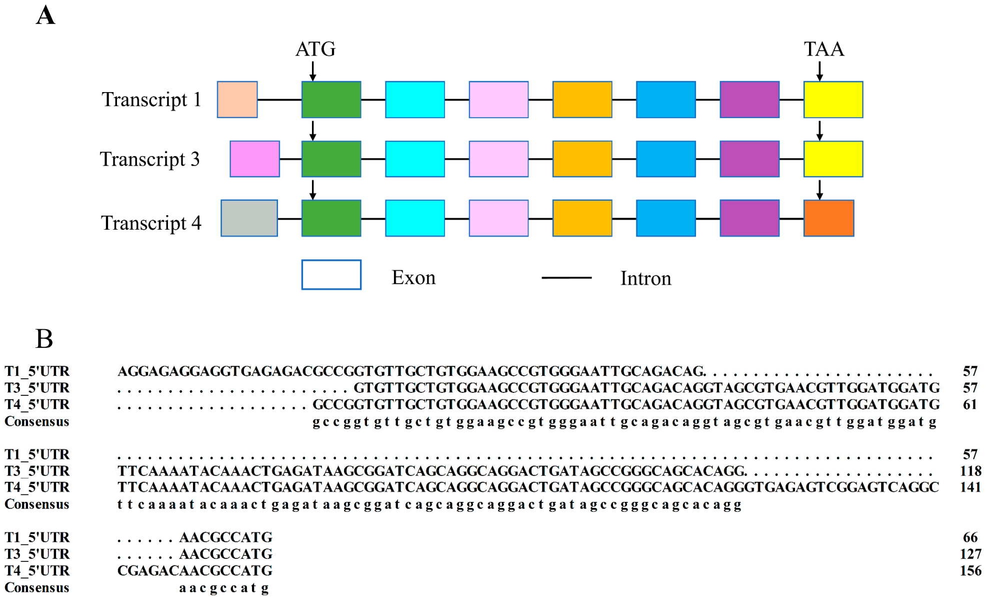

Supplemental Fig. S4. Structure and 5'UTR region differences of *DHCR7* transcripts. (A) Structural diagram of three *DHCR7* transcript variants (T1, T3 and T4). (B) Differences in the 5'UTR regions of T1, T3 and T4 of *DHCR7*.

Supplemental Tables

Supplemental Table S1 Primer sequences used in the present study. TM, Annealing Temperature.

The italic font represents protected bases, and the underscore represents the enzyme cleavage sites.

Prime	Sequence (5′-3′)	TM	Aim
DHCR7 F	ACTGACTTATACGGCTGCTGGAAT	60	RT-qPCR
DHCR7 R	GCAGGAGTGACAGCACCTTCTT		
T1 F	AGAGGAGGTGAGAGACGC	60	RT-qPCR
T1 R	ATGCTCTTCCCCACTGTG		
T3 F	CTGAGATAAGCGGATCAG	60	RT-qPCR
T3 R	TCTACCTCCCATGCTCTT		
T4 F	CAGGGTGAGAGTCGGAGT	60	RT-qPCR
T4 R	CCCCATAAGCAAGTTGATG		
ESR1 F	GCTCTCACCCTTCATCCAT	60	RT-qPCR
ESR1 R	GACATCCTCTCACGAATGC		
ESR2 F	CTGCCTGTCACCAGAGAGAC	60	RT-qPCR
ESR2 R	TTGCAGCCTTCACATGACCA		
CDK1 F	TGGCCTTGAACCACCCATAC	60	RT-qPCR
CDK1 R	AGGCAGGCAGGCAAAGATAA		
CDK2 F	CCAGAACCTCCTCATCAAC	60	RT-qPCR
CDK2 R	CAGATGTCCACAGCAGTC		
CCND1 F	ATAGTCGCCACTTGGATGCT	60	RT-qPCR
CCND1 R	AACCGGCTTTTCTTGAGGGG		
CCND2 F	TCCGGAAACATGCACAAACG	60	RT-qPCR
CCND2 R	CCGGACTTGCCTAAGGTTGC		
StAR F	TGCCTGAGCAGCAGGGATTTATCA	60	RT-qPCR
StAR R	TGGTTGATGATGGTCTTTGGCAGC		
CYP11A1 F	TGAATATCATCAGCCCCCGC	60	RT-qPCR
CYP11A1 R	GTAGGGCTTGTTGCGGTAGT		
HSD3B F	TGGAAGAAGATGAGGCGCTG	60	RT-qPCR
HSD3B R	GGAAGCTGTGTGGATGACGA		
Caspase3 F	TGGTGGAGGTGGAGGAGC	60	RT-qPCR
Caspase3 R	CCTGAGCGTGGTCCATCTTT		
Caspase8 F	GCCTTCTTCCAAGCATTACA	60	RT-qPCR
Caspase8 R	TCTCTCTCCATCTCCTCTCG		
Caspase9 F	TCCCGGGCTGTTTCAACTT	60	RT-qPCR
Caspase9 R	CCTCATCTTGCAGCTTGTGC		
GAPDH F	GAGGGTAGTGAAGGCTGCTG	60	RT-qPCR
GAPDH R	CACAACACGGTTGCTGTATC		

hRluc F	ACCAAGACAAGATCAAGGCC	60	RT-qPCR
hRluc R	GAACTCCTCAGGCTCCAGT		
hFluc F	CCATTCTTCGAGGCCAAGGT	60	RT-qPCR
hFluc R	CAGTAGGCAATGTCGCCAGA		
T4-m^{6}A F	CAGACAGGTAGCGTGAACGTTG	61	MeRIP-RT-qPCR
T4-m^{6}A R	TGACTCCGACTCTCACCCTGT		
ER-CUT F	CTGAATACGAGCCAGCAGTG	67	CUT&RUN-qPCR
ER- CUT R	TACTGAGGTGCCCAAAACTG		
SREBP2- CUT F	TGGATCAGGAGTGGTGTGGT	67	CUT&RUN-qPCR
SREBP2- CUT R	GACCTTCACAAGCCTTGTTGCC		
Spike in F	GCCTTCTTCCCATTTCTGATCC	67	CUT&RUN-qPCR
Spike in R	CACGAATCAGCGGTAAAGGT		
ESR1-CDS F	*CTA*GCTAGCATGACCATGACCCTTCACACCA	65	Overexpression analysis
ESR1-CDS R	*CCG*CTCGAGTTAT ATTGTATTCTGCATAC		
ESR2-CDS F	*GG*GGTACCGCCACCATGTCCCTCTGTGCATCTTC TCA	64	Overexpression analysis
ESR2-CDS R	*GC*TCTAGAGA CCTGGAAATG TGAAACTTGT T		
pGL3-T1-F1 F	*CGG*GGTACCCATCTCGCACTGTTTCCC	63	Promoter analysis
pGL3-T1-F1 R	*GGA*AGATCTGCGTCTCTCACCTCCTCTCC		
pGL3-T1-F2 F	*CGG*GGTACCGTGTCTTTCAGAATACAGCGG	63	Promoter analysis
pGL3-T1-F2 R	*GGA*AGATCTGCGTCTCTCACCTCCTCTCC		
pGL3-T1-F3 F	*CGG*GGTACCCTAACACACACACACATACATACG	63	Promoter analysis
pGL3-T1-F3 R	*GGA*AGATCTGCGTCTCTCACCTCCTCTCC		
pGL3-T1-F4 F	*CGG*GGTACCTGCCTGATGGAATGGGAC	63	Promoter analysis
pGL3-T1-F4 R	*GGA*AGATCTGCGTCTCTCACCTCCTCTCC		
pGL3-T1-F5 F	*CGG*GGTACCGGTTGGTCTCTTCTCACTGTTG	63	Promoter analysis
pGL3-T1-F5 R	*GGA*AGATCTGCGTCTCTCACCTCCTCTCC		
pGL3-T1-F6 F	*CGG*GGTACCACTATCCAGAAGCACCTGC	63	Promoter analysis
pGL3-T1-F6 R	*GGA*AGATCTGCGTCTCTCACCTCCTCTCC		
pGL3-T1-F7 F	*CGG*GGTACCTGGCGATGCTAATACCTG	63	Promoter analysis
pGL3-T1-F7 R	*GGA*AGATCTGCGTCTCTCACCTCCTCTCC		
pGL3-T3-F1 F	*CGG*GGTACCCATCTCGCACTGTTTCCC	63	Promoter analysis
pGL3-T3-F1 R	*GGA*AGATCTACGGCTTCCAC AGCAACA		
pGL3-T3-F2 F	*CGG*GGTACCCCAAAGTGTCTTTCAGAATACA	63	Promoter analysis
pGL3-T3-F2 R	*GGA*AGATCTACGGCTTCCACAGCAACA		
pGL3-T3-F3 F	*CGG*GGTACCCACAACCATGCTATTGGCTG	63	Promoter analysis
pGL3-T3-F3 R	*GGA*AGATCTACGGCTTCCACAGCAACA		
pGL3-T3-F4 F	*CGG*GGTACCGAATGGGACCTTGGTGTACT	63	Promoter analysis
pGL3-T3-F4 R	*GGA*AGATCTACGGCTTCCACAGCAACA		
pGL3-T3-F5 F	*CGG*GGTACC AGGAGGCTGAAGAGAGACC	63	Promoter analysis
pGL3-T3-F5 R	*GGA*AGATCTACGGCTTCCACAGCAACA		

pGL3-T3-F6 F	*CGG*GGTACCACTTAACACTTACATTCCCA	63	Promoter analysis
pGL3-T3-F6 R	*GGA*AGATCTACGGCTTCCACAGCAACA		
pGL3-T3-F7 F	*CGG*GGTACCTCTGGACCGAGCCTGTATC	63	Promoter analysis
pGL3-T3-F7 R	*GGA*AGATCTACGGCTTCCACAGCAACA		
pGL3-T4-F1 F	*CGG*GGTACCGATTGCACAGAAGA ATCCTGCTTG	54	Promoter analysis
pGL3-T4-F1 R	*GGA*AGATCTGCATTACTCCAGCTGTTCTCA GTGG		
pGL3-T4-F2 F	*CGG*GGTACCGGAATCTCACTCTCCCATC	54	Promoter analysis
pGL3-T4-F2 R	*GGA*AGATCTGCATTACTCCAGCTGTTCTCA GTGG		
pGL3-T4-F3 F	*CGG*GGTACCGATGGAATGGGACCTTGG	54	Promoter analysis
pGL3-T4-F3 R	*GGA*AGATCTGCATTACTCCAGCTGTTCTCA GTGG		
pGL3-T4-F4 F	*CGG*GGTACCTGTTGGTCTCTTCTCACTG	54	Promoter analysis
pGL3-T4-F4 R	*GGA*AGATCTGCATTACTCCAGCTGTTCTCA GTGG		
pGL3-T4-F5 F	*CGG*GGTACCGGCAAAGCCCTGACTATC	54	Promoter analysis
pGL3-T4-F5 R	*GGA*AGATCTGCATTACTCCAGCTGTTCTCA GTGG		
pGL3-T4-F6 F	*CGG*GGTACCGCTCTTGGCGATGCTAATAC	54	Promoter analysis
pGL3-T4-F6 R	*GGA*AGATCTGCATTACTCCAGCTGTTCTCA GTGG		
pGL3-T4-F7 F	*CGG*GGTACCCCGAAACGACAGACAAGG	54	Promoter analysis
pGL3-T4-F7 R	*GGA*AGATCTGCATTACTCCAGCTGTTCTCA GTGG		
ESR1 SiRNA1	GGATGTCTAGTACCAATGA		RNAi
ESR1 SiRNA2	GCCACTAACCAGTGTACTA		
ESR1 SiRNA3	CCAGTGTACGGCTCTACTA		
ESR2 SiRNA1	GTCAGACATGCAAGTAATA		RNAi
ESR2 SiRNA2	GTGCATCTTCTCACAAGGA		
ESR2 SiRNA3	GTCACCAGAGAGACATTAA		

Supplemental Table S2. SRAMP prediction results of *DHCR7* m^{6}A sites. Position: base location of the m^{6}A site in the sequence.

Seq_ID	Position	Sequence context	Score (Binary)	Score (Spectrum)	Score (Combined)	Classification
DHCR7.t1	501	GGACT	0.708	0.418	0.592	Moderate confidence
DHCR7.t1	805	GGACT	0.728	0.47	0.625	High confidence
DHCR7.t1	908	GGACT	0.734	0.461	0.625	High confidence
DHCR7.t1	1516	AAACT	0.63	0.587	0.613	Moderate confidence
DHCR7.t1	1660	AGACT	0.655	0.671	0.661	High confidence
DHCR7.t1	1738	GAACT	0.632	0.607	0.622	High confidence

DHCR7.t1	2379	GAACT	0.697	0.48	0.61	Moderate confidence
DHCR7.t3	97	GGACT	0.753	0.411	0.616	Moderate confidence
DHCR7.t3	562	GGACT	0.708	0.418	0.592	Moderate confidence
DHCR7.t3	866	GGACT	0.728	0.47	0.625	High confidence
DHCR7.t3	969	GGACT	0.734	0.461	0.625	High confidence
DHCR7.t3	1577	AAACT	0.63	0.587	0.613	Moderate confidence
DHCR7.t3	1721	AGACT	0.655	0.671	0.661	High confidence
DHCR7.t3	1799	GAACT	0.632	0.607	0.622	High confidence
DHCR7.t4	101	GGACT	0.753	0.473	0.641	High confidence
DHCR7.t4	591	GGACT	0.708	0.418	0.592	Moderate confidence
DHCR7.t4	895	GGACT	0.728	0.47	0.625	High confidence
DHCR7.t4	998	GGACT	0.734	0.461	0.625	High confidence
DHCR7.t4	1606	AAACT	0.63	0.587	0.613	Moderate confidence
DHCR7.t4	1750	AGACT	0.655	0.671	0.661	High confidence
DHCR7.t4	1828	GAACT	0.632	0.607	0.622	High confidence
